# Correspondence: Reply to ‘Revisiting the theoretical cell membrane thermal capacitance response’

**DOI:** 10.1038/s41467-017-00436-4

**Published:** 2017-11-10

**Authors:** Mikhail G. Shapiro, Kazuaki Homma, Sebastian Villarreal, Claus-Peter Richter, Francisco Bezanilla

**Affiliations:** 1 Division of Chemistry and Chemical Engineering, California Institute of Technology, 1200 E. California Blvd, Pasadena, CA 91125 USA; 2 Department of Otolaryngology—Head and Neck Surgery, Northwestern University, 303 E. Chicago Ave, Evanston, IL 60611 USA; 3 The Hugh Knowles Center for Clinical and Basic Science in Hearing and its Disorders, Northwestern University, Evanston, IL 60208 USA; 4 Department of Biochemistry and Molecular Biology, University of Chicago, 929 E. 57th Street, GCIS W244, Chicago, IL 60637 USA; 5 Department of Biomedical Engineering, Northwestern University, 2145 Sheridan Rd, Evanston, IL 60208 USA; 6 Department of Communication Sciences and Disorders, Northwestern University, 2240 Campus Dr, Evanston, IL 60208 USA

## Introduction

We thank Plaksin, Kimmel, and Shoham for their correspondence regarding our 2012 article on the mechanism of infrared stimulation of excitable cells^1^. In this study, we showed that the heating of cellular water by infrared light leads to an increase in the electrical capacitance of the cell membrane. This time-varying capacitance produces a current leading to membrane depolarization and generation of action potentials. Although our experimental findings were the primary focus of the paper and account for most of its impact to date, we also attempted to provide a theoretical explanation of how the membrane capacitance changes with temperature.

As Plaksin et al. point out in their accompanying correspondence, our theoretical explanation relied on the Genet et al.^2^ model of the coupled double layer capacitance across the cell membrane. In adapting this model, we did not account for a difference between Genet’s sign convention for transmembrane charge and what is typically used in electrophysiology studies. After correcting for this difference, it is clear that the suggested theory does not explain our experimental findings. Although the distribution of mobile charges on each side of the bilayer does change with temperature, the net effect of these changes is predicted to decrease, rather than increase, the apparent bilayer capacitance. Therefore, alternative theories are needed to provide a complete understanding of thermal stimulation. For example, Plaksin et al.^3^ have proposed a complete theory that considers recent experimental measurements of bilayer thickness as a function of temperature^4^.
